# Supplementary material for: Bioinformatics-Based Analysis of Key Genes in Steroid-Induced Osteonecrosis of the Femoral Head That Are Associated with Copper Metabolism
Source: Biomedicines. 2023 Mar 13;11(3):873. doi: 10.3390/biomedicines11030873 (PMC10045807; doi:10.3390/biomedicines11030873)
Supplement: Supplementary file 1 [file biomedicines-11-00873-s001.zip › Supplementary Figues S1-S3.pdf]

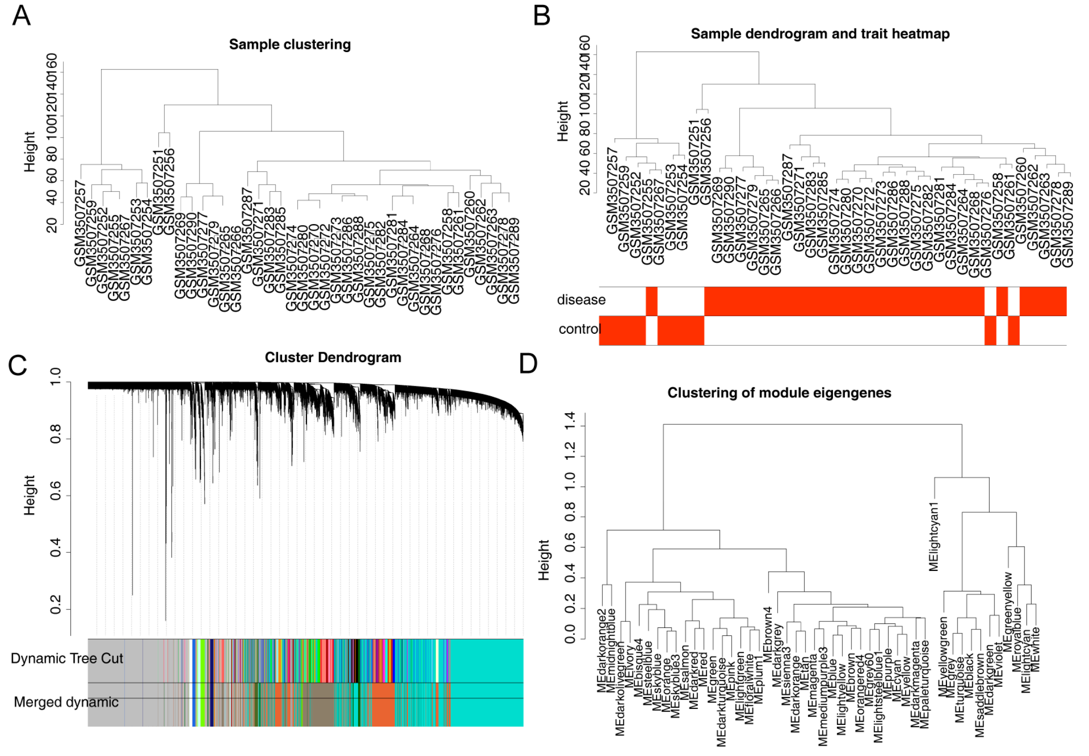

Figure S1: WGCNA analysis. (**A**) Overall clustering of the samples from the dataset; (**B**) The samples were classified, and heatmaps showing the sample clustering and clinical characteristics were constructed; (**C**) Cluster dendrogram of genes in the coexpression network; (**D**) Cluster dendrogram of samples.

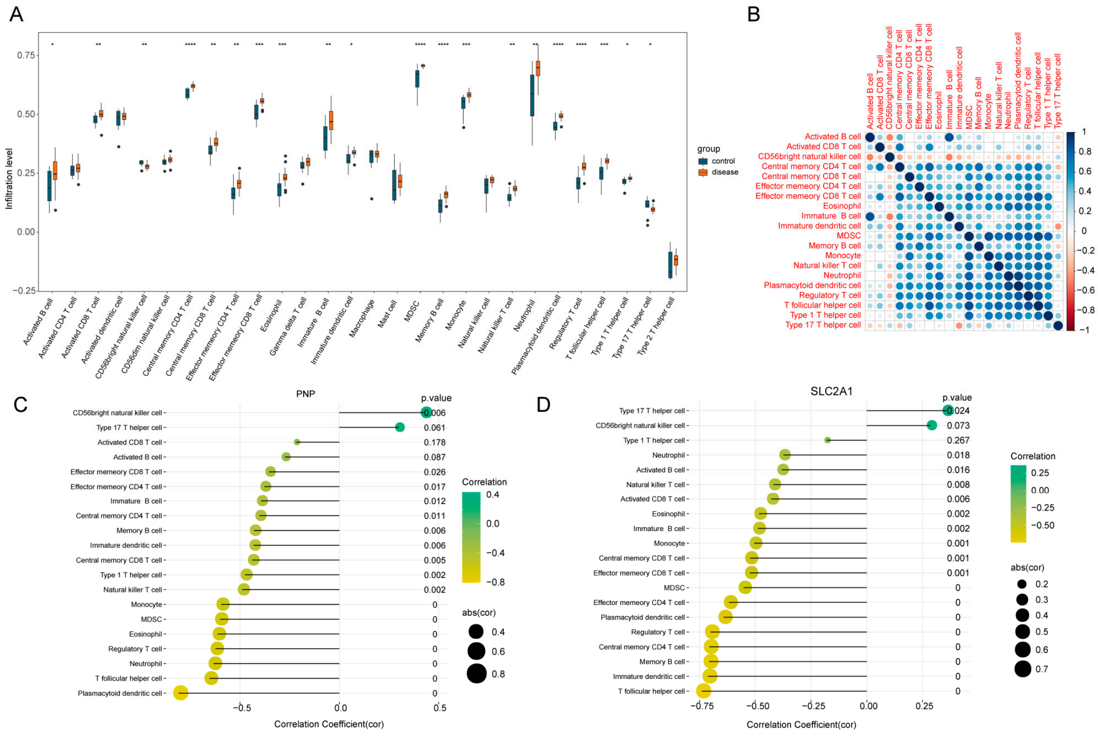

Figure S2. Abundance of infiltrating immune cell populations in all samples between the

SONFH and control groups. (**A**) Twenty immune cell populations had significantly different abundances between the SONFH and control samples; (**B**) The correlation among differentially abundant cells was calculated; (**C**) The correlation between PNP expression and differential cell population abundance; (**D**) The correlation between SLC2A1 expression and differential cell population abundance. * $p < 0.05$, ** $p < 0.01$, *** $p < 0.001$,**** $p < 0.0001$

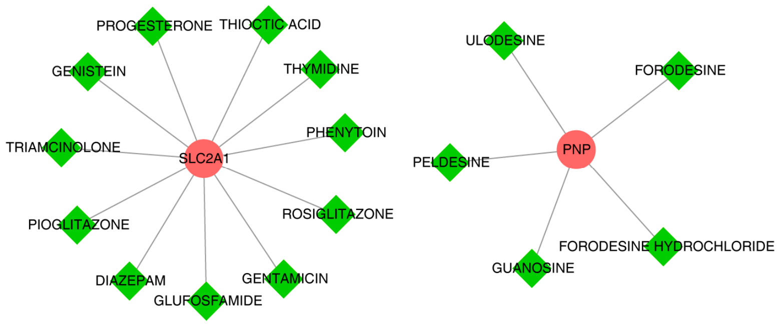

Figure S3. Sixteen drugs in the DGIdb database were predicted to target PNP and SLC2A1. Red circle repreasents key gene, and green diamond repreasents targeted drugs.
